# Biological versus Clinical Risk Factors in Acute Myeloid Leukemia: Is There a Winner?

**DOI:** 10.1155/2019/3914828

**Published:** 2019-06-11

**Authors:** M. Malagola, N. Polverelli, V. Cancelli, E. Morello, A. Turra, E. Borlenghi, F. Cattina, B. Rambaldi, S. Bernardi, C. Zanaglio, Elif Dereli Eke, L. Gandolfi, M. Farina, D. Russo

**Affiliations:** ^1^Chair of Hematology, Department of Clinical and Surgical Sciences, University of Brescia, Bone Marrow Transplant Unit, ASST-Spedali Civili, Brescia, Italy; ^2^Unit of Hematology, ASST-Spedali Civili, Brescia, Italy; ^3^Centro di Ricerca Ematologica-Oncologia AIL (CREA), ASST-Spedali Civili, Brescia, Italy

## Abstract

We present a case of a patient with a three-month history of peripheral blood cytopenia without a confirmed diagnosis of myelodysplastic syndrome, who developed a favourable-risk acute myeloid leukemia (AML), according to the European Leukemia Net (ELN) criteria. The patient achieved a complete remission with incomplete platelet recovery (CRi) after induction. The patient achieved the morphological CR after the first consolidation and completed the first-line treatment with a syngeneic stem cell transplantation (SCT). A disease relapse occurred after one year of CR (blast cell count in the bone marrow 15%), and the patient was offered a haplo-SCT, which he refused due to personal reasons. In this paper, we discuss the interplay between clinical and biological risk factors in non-high-risk AML patients and speculate that some old clinical risk factors (e.g., age of the patient, achievement of CR after induction, and previous history of myelodysplastic syndrome) may still impact on the treatment decision algorithm of some of these patients.

## 1. Introduction

The treatment algorithm of newly diagnosed AML patients is currently driven by ELN criteria [1]. ELN classification is based on the presence of specific mutations on target genes, with the identification of peculiar clinical-biological entities (e.g., AML with FlT3-ITD mutation or NPM-1A mutation, variably combined together). According to these criteria, allogeneic stem cell transplantation (allo-SCT) should be offered to those patients aged less than 65 years, who have less than 20–30% of expected long-term overall survival with conventional chemotherapy (usually accounting for 40–50% of the cases). Favourable and intermediate risk patients, who may be cured in 50 to 80% of the cases with conventional chemotherapy, are usually excluded from first-line allo-SCT. In this setting, there is still a debate on the role of some clinical risk factors (e.g., patient's age, previous history of myelodysplastic syndrome, and CR achievement after induction), and the management of these patients is still largely dependent on single-center internal guidelines.

## 2. Case Presentation

Recently, a 67-year-old man with acute myeloid leukemia (AML) was referred to our Transplant Center for salvage treatment in overt relapse. He was diagnosed with a primary AML in December 2017. A three-month history of peripheral blood cytopenia before AML diagnosis was present, without a confirmed diagnosis of myelodysplastic syndrome. The cytogenetics was normal; Flt3-ITD and point mutation and NPM mutation were absent; the only molecular lesion as detected by RT-qPCR was a biallelic CEBP-alpha mutation. This lesion was absent in the germinal DNA. Thus, the disease risk was classified as favourable according to the recently published European Leukemia Net (ELN) criteria [1]. The patient received an induction with a conventional idarubicine, cytarabine, and etoposide combination and achieved a complete remission with incomplete platelet recovery (CRi). Subsequently, 3 cycles of high-dose cytarabine was administered. Considering the standard risk of the disease at diagnosis, the availability of a homozygous twin, and the expected low transplant-related mortality (TRM) of syngeneic allo-SCT, the latter was performed for intensification. The conditioning regimen was adapted to the patient's age (busulfan 6.4 mg/kg total dose and fludarabine 160 mg/sqm total dose). A complete hematological recovery was obtained after the first consolidation cycle and maintained since November 2018, when peripheral blood cytopenia occurred, with a bone marrow leukemic infiltration of 15%. Thus, the patient was referred for salvage treatment with haplo-SCT from his son. Unfortunately, the patient refused the procedure due to personal reason and is currently lost to follow-up.

## 3. Discussion

In the nineties, the prognosis of AML was mainly influenced by cytogenetics and by clinical risk factors, mainly represented by the age, the achievement of CR after the first induction cycle, and a previous history of myelodysplastic syndrome [2]. In those years, the decision of whether or not to perform an allo-SCT was relatively easy to take, and 60–70% of the cases were addressed to allo-SCT as soon as possible. In the subsequent years, the biological characterization of AML underwent a rapid and progressive evolution, and now, more than 25 genes have been clearly identified as crucial for AML onset, progression, and prognosis [3]. Among these genes, the most important ones for disease-risk identification are Flt3, NPM-1, IHD-1, IDH-2, CEBP-alpha, GATA-1, ASXL1, and P53 [3]. As a consequence, the recently published ELN criteria for AML risk stratification in AML include some of these genes and identify peculiar biological entities, with different prognosis and for which different therapeutic strategies are suggested [1]. Moreover, minimal residual disease (MRD), either by flow cytometry or by RT-PCR on target genes, has been integrated in the dynamic assessment of treatment response, thus refining risk allocation [4–9]. A recent paper published by Chen and colleagues suggested that both MRD and the type of response after induction chemotherapy (CR vs CRi) are independent prognostic factors for outcome in patients achieving remission [6]. Minetto and colleagues addressed the issue of the best time-point of MRD assessment by flow cytometry. In a cohort of AML patients treated with a fludarabine-based induction regimen, they showed that early MRD assessment (after induction) is the strongest predictor of outcome [8]. Moreover, they confirmed their previous observation that the integration MRD monitoring by flow cytometry and molecular biology on WT1 leads to an improvement in prognostic stratification. This was previously demonstrated in their paper on the impact of MRD status before allo-SCT. Thanks to the combination of the high positive predictive value of WT1 MRD and the high negative predictive value of flow cytometry MRD, three cohorts of patients could be identified according to MRD status before transplant, showing worsening outcome: negative by WT1 and flow cytometry, positive by flow cytometry and negative by WT1, and positive by WT1 and flow cytometry [8]. In this complex scenario, allo-SCT should be offered to those patients who have less than 20–30% of expected long-term overall survival with conventional chemotherapy (usually high-risk AML) [7]. In the favourable/intermediate-risk group, the old clinical prognostic factors should be considered in the treatment algorithm, with particular regard to allo-SCT indication. In this setting, it is unquestionable that a 35-year-old man with a primary resistant AML should be addressed to allo-SCT as soon as a CR is achieved, but the agreement among hematologists of whether or not to perform an allo-SCT front-line in a patient as the one reported here is probably less evident (67-year-old patient, with favourable risk AML, with CRi after induction, and with a previous history of peripheral blood cytopenia). This fact may reflect the enthusiasm of hematologists in the era of molecular markers of AML that put a shadow on the role of the old clinical factors. In the clinical case presented, the choice to perform a syngeneic SCT was made, considering the safety of the procedure even though it is well known that the lack of graft versus leukemia (GVL) effect in this transplant is the cause of its failure in the great majority of patients. Moreover, the reduction of the conditioning intensity, which was necessary for the patient's age, is often performed in the setting of allo-SCT, where the GVL effect is present. In the setting of syngeneic SCT, the reduction of conditioning intensity put the patient at risk of disease recurrence, for incomplete leukemia eradication. How could we improve the risk definition of the patient presented in this case? The recent development of the next generation sequencing (NGS) technology has dramatically expanded the genes that can be studied and analyzed both in the diagnostic phase and in the MRD monitoring [10]. An extensive study of the genes involved in leukemogenesis and with known prognostic impact could help in better depicting the complexity of the leukemic transformation and could give clear prognostic information. Focusing on this clinical case, if we had found mutations in genes such as p53 or DNMT3A or ASXL1 at diagnosis, we would have considered the patient at high risk of disease recurrence, thus with a strong indication of front-line allo-SCT. The extensive and routine use of NGS technology in the incoming future will be a useful tool to improve the prognostic classification of AML.

In 2011, we published a simple and reproducible prognostic score for cytogenetically normal AML (CN-AML) [11]. This score was created in 337 patients with CN-AML (training set) and then validated in a cohort of 197 CN-AML (validation set). The variables that were independent prognostic factors for event-free survival (EFS) and overall survival (OS) in the training set were: age ≥ 50 years, secondary AML, and white blood cell count (WBC) ≥ 20 × 10^9^/L. The patients of the training set were stratified into three groups: low, intermediate, and high risk. The median EFS was 25, 12, and 7 months in the low-, intermediate-, and high-risk groups (*p* < 0.0001), respectively. The median OS was not reached in the low-risk group and was 19 and 10 months in the intermediate- and high-risk groups (*p* < 0.0001). Very similar results were obtained in the validation set. We concluded that the score was useful for dissecting patients with CN-AML and different prognosis and that it could be integrated with the biological markers that, in those years, were still not completely validated.

**Figure 1:**
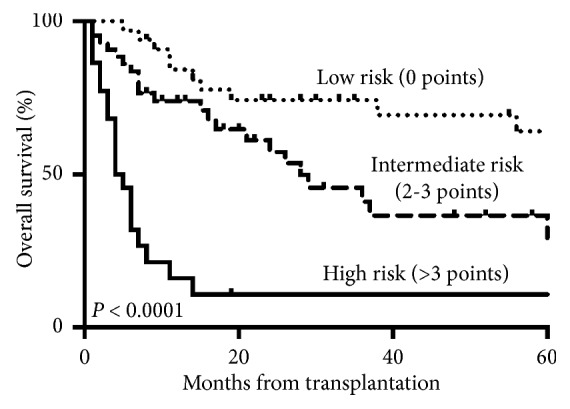
OS of the 101 patients with AML submitted to allo-SC, according to the PIS.

We recently retrospectively collected the data on 101 AML patients submitted to allo-SCT in our Center, between 2010 and 2017. In about 50% of the cases, the median age was greater than 55 years and 79% of the patients had a primary AML. Focusing on ELN risk, 60% and 40% had an unfavorable and a favourable/intermediate-risk AML, respectively. The patients with favourable/intermediate-ELN risk were addressed to allo-SCT because of primary refractory AML in 69% of the cases and for other clinical risk factors in 31% of the cases. 53% of the patients were allotransplanted in first CR, 55% received a myeloablative conditioning regimen, and 73% received peripheral blood stem cells. 43% and 44% of them received a matched sibling and a matched unrelated donor, respectively. At the time of allo-SCT, the HCT-CI index according to the published criteria [12] was <3 in 36% of the cases and the performance status according to the Karnofsky index was 100% in 40%, between 80 and 90% in 51%, and less than 70% in 9% of the cases. By univariate analysis, the factors significantly influencing OS were age >55 years at transplant (*p*=0.037; HR 1.77; 95% CI 1.04–3.01), induction refractoriness (*p*=0.003; HR 2.47; 95% CI 1.37–4.44), morphological CR at allo-SCT (*p* < 0.001; HR 0.25; 95% CI 0.14–0.45), and Karnofsky PS < 90% (*p* < 0.001; HR 3.59; 95% CI 2.96–6.26). By multivariate analysis, the independent factors associated with OS were age >55 years at transplant (*p*=0.03; HR 2.3; 95% CI 1.33–4.00), morphological CR at allo-SCT (*p*=0.039; HR 0.45; 95% CI 0.21–0.96), and Karnofsky PS < 90% (*p*=0.01; HR 2.56; 95% CI 1.22–5.36). A prognostic index score (PIS) was calculated by totaling the score derived from the regression coefficients of each clinical variable significantly associated with prognosis by multivariate analysis. In particular, the following scores were associated to the single variables: 2 for age >55 years, 3 for Karnofsky <90%, and 2 for no CR at the time of allo-SCT. Patients were grouped in low-risk (PIS = 0), intermediate-risk (PIS 2-3), and high-risk (PIS > 3) groups.  Figure 1 reports the OS of patients in the three cohorts. The OS at 5 years was 65%, 35%, and 10% in the low-, intermediate- and high-risk groups, respectively (*p* < 0.0001).

Our data, both in patients with CN-AML [11] and in patients submitted to allo-SCT (unpublished), strongly suggest that clinical factors still have an impact on the outcome of AML. We think that these factors should still be accurately integrated in the risk assessment of AML. In the era of the next generation sequencing, many more data on molecular markers of AML will be available [10]. Moreover, the dynamic monitoring of MRD either by flow cytometry or by molecular biology is becoming a useful tool for early allocation of patients in different risk categories and, for example, for addressing patients who do not clear MRD to intensive treatments or new target drugs before allo-SCT [8, 9]. As a consequence, clinicians have to be very skillful in combining these data with the clinical prognostic factors. In other words, we think that there is no winner between biology and clinic, but a strong integration between these two elements is helpful to choose the best treatment program for our patients.
